# Therapists’ Experiences of Psychodynamic Therapy with and without Transference Interventions for Adolescents with Depression

**DOI:** 10.3390/ijerph17134628

**Published:** 2020-06-27

**Authors:** Maria Jones, Marit Råbu, Jan Ivar Røssberg, Randi Ulberg

**Affiliations:** 1 Department of Psychology, University of Oslo, Pb. 1094 Blindern, 0317 Oslo, Norway; mariraa@psykologi.uio.no; 2 Institute of Clinical Medicine, Faculty of Medicine, University of Oslo, 0318 Oslo, Norway; j.i.rossberg@medisin.uio.no; 3 Division of Mental Health and Addiction, Oslo University Hospital, 0407 Oslo, Norway; randi.ulberg@medisin.uio.no; 4 Department of Psychiatry, Diakonhjemmet Hospital, Diakonveien 12, 0370 Oslo, Norway

**Keywords:** psychodynamic psychotherapy, adolescent therapy, depression, qualitative methods, therapist interviews

## Abstract

Psychodynamic therapy is an effective treatment for depression. However, a large number of adolescent patients with depression do not respond and/or drop out of therapy and little is known about what therapists actually do in therapy with adolescents. Thus, more research is needed to explore the various actions that therapists do in therapy, so that therapists can tailor their therapy more specifically to each individual adolescent. The present study aimed to investigate how the experience of psychotherapists differs between two treatment modes for adolescents with depression: psychodynamic psychotherapy with and without transference interventions. In-depth interviews were conducted with six therapists. The data, which were analyzed using thematic analysis, generated three key themes: (1) The therapists experienced that transference interventions are often useful in therapies with adolescents with depression, (2) therapies without transference interventions can be challenging for therapists, but still helpful for patients, and (3) the experience contributed to the deepening recognition of therapists that they always need to adapt their techniques to the particular patient. The results enhance our knowledge of the significance of therapists’ actions in therapy with adolescents. The therapists highlighted issues that are important for identifying barriers to incorporating new knowledge into clinical practice.

## 1. Introduction

Depression is a common mental health problem for adolescents, worldwide, with an estimated one-year prevalence rate of 4–5% in mid to late adolescence [1]. Studies suggest that the prevalence of depression in adolescents, especially females, has increased in recent years [2,3,4]. In addition to causing distress in an individual’s life, depression during adolescence and young adulthood also increases the risk of psychological, psychosocial, and somatic problems in later life [5,6,7,8].

There is little evidence for the effectiveness of depression prevention programs in adolescence [9,10,11,12]. Psychotherapy is the preferred treatment for adolescents with depression [13]. Systematic research, including randomized controlled trials (RCTs), has shown that psychodynamic or psychoanalytic therapy promotes long-lasting therapeutic change in adults and youth [14,15,16,17,18,19,20]. However, a recent meta-analysis has underscored the need for improved therapies, especially for adolescents with depression [21]. Some 40–50% of children and adolescents with depression are not responsive to treatment, with dropout rates of 40–60% [22,23]. Tailoring treatment more specifically to each patient may be necessary to reduce non-responsiveness and dropping out, as one treatment does not fit all [24]. We need to know what kind of interventions are helpful for each young person in order to personalize treatment [25]. Systematic research on specific interventions in psychodynamic therapy with adults has revealed that some patients profit more from working with transference than others according to various patient characteristics [26,27,28,29,30]. Transference interventions (TI) seem to have specific positive effects on long-term functioning in patients with personality problems, as well as serious and chronic difficulties in establishing stable and meaningful relations [26,27,28,30]. Patients with higher relational functioning seem to benefit from low to moderate levels of TI, but they have difficulty with high levels of TI [26,27].

While there are many areas of overlap between depression in young people and adults, there are also significant differences. Adolescents are at a different developmental level than adults and usually live with caregiver(s). Longitudinal data indicate that low perceived family support and school connectedness is associated with greater depressive symptoms among adolescents [31]. Several other aspects of their life, such as the possible stress of living at home, how family members interact, and their socioeconomic status can influence the adolescent’s psychophysical state of health, and this may be something that youths cannot escape from or change [32,33]. Thus, there are important differences between therapy with adolescents and adults [34]. Studies on psychotherapy with youths indicate that outcomes are related to the way a therapist relates to patients. The therapist’s capacity to be non-judgmental and offer empathy, warmth, genuineness, honesty, flexibility, involvement, guidance, and instruction are particularly important [35,36,37]. Less is known about how the therapist’s skills and the therapeutic relationship affect a helping relationship [38].

A qualitative inquiry is seen as the primary choice to study experiences, thoughts, expectations, motives, and attitudes processes—and the “how” and “what” questions of process and change [39,40]. More recently, mixed-methods approaches, such as including (“nesting”) qualitative studies in RCTs, has also received attention as a way to more fully understand the impact of therapeutic interventions within complex clinical settings [41]. Previous mixed-method designs have focused on client perspectives [41,42,43,44,45]. Fewer have focused on the clinicians’ perspective, even though investigators have found characteristics of the clinician to be critical for the adoption of evidence-based practices (EBP) [46,47].

Qualitative interview studies with clinicians play a key role within the broader therapy research literature. The field has evolved from a need for sharing practical knowledge, and is an alternative perspective to findings of other types of research, such as RCTs or qualitative studies of client experiences [48,49,50,51]. Study designs that capture different dimensions of professional knowledge include first-person autobiographical and auto-ethnographic studies that present the accumulated professional knowledge of an individual clinician in relation to a specific client group [52,53] or a phase within a career [54]. Other studies have investigated the experience and knowledge of clinicians with respect to discrete areas of practice [55,56,57] or sought to reflect the totality of professional experience [49]. The present study does not focus on a clinical field itself (e.g., self-harm or partner violence), but rather, specific interventions that are used when working with a specific patient group (adolescents with depression). Insight into the techniques typically used by therapists working with depressed adolescents is an area that little is known about [58]. This paper seeks to reduce this knowledge gap and enhance the understanding of TI in psychodynamic therapy with adolescents, from the therapists’ perspective. The aim was to explore how therapists experienced therapies with and without transference interventions for adolescents with depression.

## 2. Methods

### 2.1. Participants

In total, six therapists were included in the study (3 males and 3 females), aged 54–71 years (mean = 63 years). All the therapists had already been recruited as therapists in the randomized clinical trial (RCT) First Experimental Study of Transference Work—In Teenagers (FEST-IT) [59]. The study had a dismantling design and aimed to explore the effect of a specific technique, transference interventions, in therapy with adolescents with depression. Patients, aged 16–18 years, who were referred to public child and adolescent outpatient clinics with a diagnosis of major depressive disorder (MDD), as defined in the Diagnostic and Statistical Manual of Mental Disorders, 4th Edition (DSM-IV), were recruited in the RCT. 70 patients were randomly assigned to psychodynamic therapy with (*N* = 39) or without (*N* = 31) transference work. One patient withdrew from the study. The allocation to treatment of patients admitted to the trial was achieved by setting up clusters of four patients for each therapist and using lottery to allocate patients to one of the two treatment groups. For each of four patients randomized to each therapist, two patients were treated with transference work and two without. The FEST-IT involved short-time psychodynamic psychotherapy (STPP), offered 28 sessions of therapy, and used the published manual of the IMPACT-study [16]. Patients were randomly assigned to two treatment groups, both of which used general psychodynamic techniques: a moderate level of TI versus no TI [59].

Interventions in psychodynamic therapy can be conceptualized on an expressive–supportive continuum. Transference interventions can be seen as expressive interventions, that can take place in many forms [60]. TI, as defined here, includes any intervention that focuses on the patient’s relationship and experience with the therapist [61,62,63]. In the transference group, therapists encouraged exploration of the patient–therapist relationship (TI). In the comparison group, therapists avoided directly focusing on the patient–therapist relationship, and instead, focused on exploration of the patient’s relationships outside therapy [64]. These relationships could be everyone in the patient’s life (friends, relatives, colleagues, classmates, etc.), defined here as relational interventions (RI) [64]. Rated on a 5-point Likert Scale (0: not at all, 4: very much), the average transference work score was 2.47 (standard deviation (SD) = 1.0) in the transference group and 0.2 (SD = 0.3) in the non-transference group (t = −7.7, degrees of freedom (df) = 18.0, *p* < 0.0005). Intraclass correlation (ICC) among raters was 0.94. The average ICC between the two raters and the randomization were 0.94. Follow-up was conducted at post-treatment and one year after treatment termination.

The therapists were either specialists in child and adolescent psychiatry, or specialists in clinical psychology with additional education in psychotherapy with adolescents. Four of the therapists were psychiatrists and two were clinical psychologists. The education of both types of therapists included a six-year program of professional studies in either medicine or psychology. All six therapists identified their theoretical affiliation as psychoanalytic/psychodynamic, and they had at least 2 years of formal training in psychodynamic therapy. They were further trained in a 1-year program to provide psychodynamic therapy with or without transference interventions. The number of patients per therapists in the present study ranged from 4 to 12 patients (mean = 7).

### 2.2. Procedure

The present qualitative study is an independent research project nested in the original RCT, FEST-IT. The inclusion criteria were therapists who had participated in the FEST-IT and had patients within the last two years (at the beginning of recruitment). Out of the nine therapists involved in the original RCT, eight met the inclusion criteria, and six were included in the study. The recruitment practice was purposive in that the participants who were recruited were considered best suited to answer or illuminate our research question [39]. All the therapists involved in the RCT were contacted individually. They were given information about the purpose of the study and were told they could pause or stop their participation at any time. The interviews were conducted by the first author. All the therapists were interviewed face to face, one at a time. The interviews were audio-recorded and lasted between 50 and 81 min (mean = 60 min).

### 2.3. Variables and Instruments

The interview guide was semi-structured and included follow up-questions. Semi-structured interviews are a recognized method for collecting qualitative data. The technique allows for the collection of consistent data across participants, as well as more in-depth examination of individual experiences [39,65,66,67]. The interview guide consisted of three main sections: (1) a section addressing the therapists’ experiences of mechanisms of change in psychodynamic therapy, in general, when working with adolescents with depression, (2) a section addressing the therapists’ experiences of transference work as a specific intervention in therapy with adolescents who had depression, and (3) a section addressing the therapists’ experiences and understanding of negative outcomes or absence of improvement in their own therapies. Examples of questions were, “Can you please describe how you experienced working with transference in the therapies?”, “How did you experience refraining from transference interventions?”, and “Did you have any therapies where you experienced that the patient did not improve, or drop out of therapy? If so, can you describe the particular therapy?” The therapists were encouraged to provide concrete examples from their own therapies. The order of the questions was handled in a flexible way, to encourage associations and important themes that were arising from the participants, while making sure that all the questions were touched upon during the interview.

The study is informed by phenomenological and hermeneutic principles. This implies acknowledging human experiences as valid knowledge, as well as the recognition that interpretation of a text is always contextual and influenced by the researcher’s background and assumptions [39,68]. The aim, first and foremost, was to explore how the participants understood and made sense of their own experiences [69].

### 2.4. Data Analysis

The data analysis was guided by the principle that themes reflect “a pattern of shared meaning, organized around a core concept or idea, a central organizing concept” [70]. Themes are built from smaller meaning units containing related data fragments [71].

The data analysis was performed based on the six phases of thematic analysis (TA), suggested by Braun et al. [70,71,72].

Step one—familiarization: The audio-recorded interviews were transcribed verbatim by the interviewer after all the interviews were conducted. A result of this is that information obtained or ideas that arose from transcribing the interviews did not influence choices during the interview process. The transcription process resulted in a total of 145 pages of transcripts. After reading and re-reading the transcripts, the first author removed text fragments unrelated to the research question, to create densified transcripts. These were read by all authors, in line with recommendations that several researchers read the transcripts to stimulate an analytic process with several nuances [39]. All authors gave feedback on their initial thoughts, before proceeding to the next analytic step. Step two—generating codes: Meaning units were identified, coded, and sorted by identifying fragments of the transcribed data material that shed light on different dimensions of the research question. This initial analysis was conducted by the first author and discussed with all the authors. Step three—constructing themes: The meaning units were grouped into preliminary themes. Some meaning units were placed into more than one theme, as they could highlight different aspects of the research questions. Step four—revisiting themes: Themes were revisited several times, and some subthemes with fewer meaning units were merged into broader and more nuanced subthemes. For example, an initial subtheme named “transference interventions can repair ruptures” was placed in the broader subtheme “working with transference strengthens the therapeutic relationship”. Meaning units that were initially placed into more than one theme were reviewed and placed in the most accurate theme. Step five—defining themes: Meaning units were then extracted and condensed into interpretative descriptions. Authentic, illustrative citations were kept and interpreted to stay true to the participants’ descriptions. During this process, the themes were renamed and defined to become more descriptive of the therapists’ experiences. For example, a theme originally defined as “challenges for the therapist” was renamed to “refraining from transference interventions can be experienced as a limitation for the therapist”, as this was regarded as more descriptive and closer to the therapists’ voices. Step six—producing the report: During this step, the transcripts and densified transcripts were revisited to review whether the themes worked individually, in relation to the dataset and overall.

The representativeness of the findings and the recurrence of the themes were indicated by frequency labels, as suggested by Hill et al. [67]. The general label describes a theme that was applied to all or all but one case and is referred to in the text as all therapists. This applies to all the three key themes. Themes were defined as typical when they applied to more than half the cases, which are referred to in the text as most therapists. Variations within themes are reported as some therapists, or the number of therapists who illustrated the subject matter (one or two therapists). Views and citations from all the therapists have been included in the results to ensure a balanced representation of the interviews and bring forward vivid illustrations of the meaning conveyed in the material [36].

### 2.5. Ethics

The study was approved by and registered in the Central Norway Regional Committee for Medical and Health Research Ethics (REC). All the participants were given a consent form to read and sign before beginning the interviews. Audio files and transcribed material were stored in a database specifically developed for sensitive data. Private details about the participants were altered to ensure their anonymity.

## 3. Results

The analysis generated three key themes that reflect different aspects of how the therapists experienced psychodynamic therapies with and without transference interventions, each of which contained two subthemes, as presented in Table 1.

### 3.1. Key Theme 1: Transference Interventions Are Often Useful

All the therapists said that, in most cases, they thought it was useful to work with transference in therapies with adolescents with depression. Two dimensions were highlighted: how they experienced that working with transference, for the most part, strengthened the therapeutic relationship, and how TI can contribute to increased insight and self-esteem.

*Subtheme (I): Working with transference strengthens the therapeutic relationship*

Most therapists said that using TI strengthened the therapeutic relationship. One therapist described the following: “There is an attention to our relationship, and I believe that it is a quite strong experience that someone is interested in how it is for you to be with me” (T1). Her experience was that this focus and joint attention in the sessions empowered the relationship. One of the therapists said that he often picked up the thread from the last session and asked adolescents how it felt for them to come back and talk to him that particular session: “I felt like they took it as an invitation to open up and that it created this proximity in the session, that we came closer to the things that were meaningful to the patients” (T3). This quote illustrates a point that all the therapists touched upon, namely that the use of TI both influenced what was talked about during therapy, as well as how they talked about topics.

The therapists highlighted how using TI can invite adolescents to address issues, worries, or thoughts they have about the therapeutic relationship or the therapy. One therapist stated the following: “It can be useful to address our relationship because they [patients] might have been afraid to do so, but they may have communicated something by coming late to sessions or had the feeling that things do not work here either” (T2).

Some therapists talked about how the quality of the therapeutic relationship depended on how the adolescents experienced the therapist. As TI provide a way to shed light on the therapeutic relationship and invite the adolescents to give their perspective about the therapeutic process, working with transference can also create an opportunity to repair ruptures in the therapeutic alliance:

*We can talk about how what I say affects the patient. If the patient says “it seems like you did not pay attention” or “you seemed critical,” I can reply that “that sounds like a tough experience and I don’t want it to be that way”. And then we ... can take a look at what happened*.

(T2)

The statement illustrates how TI can be used to invite the patient to address her thoughts and needs. The therapist also mentioned how his response and acknowledgement of the patient’s experience can help an adolescent acknowledge and accept other people’s limitations. One of the therapists explicitly addressed that it was important [for therapists] to be mindful of the available time when they consider interventions:

*Patients may become more attached when I use transference interventions and that can be for better or worse, especially in a short-term therapy. The therapeutic relationship gets closer and more vulnerable when I open up for addressing and commenting on our relationship. And then, all of a sudden, it is time to end [the therapy]*.

(T5)

As TI were considered techniques that can strengthen the therapeutic relationship, this quote clearly suggests that therapists should be cautious about what interventions they use given a limited amount of time, especially near the end of a therapeutic process.

*Subtheme (II): Transference interventions can contribute to increased insight and greater self-esteem*

This theme involved the belief that TI can contribute to increased insight, greater self-esteem, and help build adolescents’ sense of agency. The therapists said TI could provide adolescents with insight by addressing what was happening between the therapist and adolescent patient, here-and-now, in the therapy room. One of the therapists described this as a *eureka moment*, when the adolescents understood that their relational patterns also played out between them and the therapist:

*When the pattern is addressed here and now, and in relation to me, it is like something changes. They realize that it is actually something they have a part in, something that they have projected onto the other [but] does not necessarily have to do with the other*.

(T5)

When relational patterns that cause distress to adolescents are played out in the therapy room, the therapists saw TI as a technique that could facilitate insight into the adolescents’ active role in the process of repeating these patterns. The therapists’ said that TI can provide adolescents a sense of agency by giving them an opportunity to reflect about themselves and having the experience of being able to control their actions and reactions. One of the therapists described it this way:

Individuals with depression often experience [an] outside world where things just happen and everything is terrible and destructive … or it is difficult to explore what is difficult and painful, so that you take the whole blame yourself. It becomes actualized in the therapy and we can point at it and describe it. So, it becomes very clear for the patient, as well. “I do the same here as I do elsewhere.”

(T6)

In these cases, TI could help adolescents gain insight into their depressive patterns. A different therapist reflected upon how TI could promote and confirm the patient’s autonomy and agency, as he changed his interventions from being more controlling in the beginning of therapy:

*… Eventually I became more confirming of her feelings, and she started to open up more and became more autonomous. Then I affirmed her autonomy and by the end of the therapy she had solved her fundamental problem, namely that she tried to be liked by everyone and did what she thought others wanted her to do*.

(T2)

This statement illustrates how the therapist experienced that the therapeutic relationship developed as he changed his interventions with the patient. In his experience, this changed how the patient related to him and others.

### 3.2. Key Theme 2: Therapies without Transference Interventions Can Be Challenging for the Therapist, but Still Helpful for the Patient

Most of the therapists said therapy without TI was challenging to some extent. Their reflections on this treatment mode could be sorted into two subthemes: (1) that refraining from TI can be experienced as a limitation for the therapist, and, however, (2) that relational interventions can be useful to analyze the patients’ lifeworld.

*Subtheme (I): Refraining from transference interventions can be experienced as a limitation for the therapist*

There was some variation in how refraining from TI was experienced. The therapists that found it challenging described a subjective experience of being constrained and restricted from being truly intuitive in their therapeutic interventions when meeting the young person. Refraining from TI was experienced as particularly challenging in cases where they sensed that the therapeutic alliance or the therapy itself was at risk:

…When there was so much going on in the therapy room that had to do with the patient’s transference towards me, and then I had to place it out there in the real world, on parents, friends, partners… It was frustrating not being able to point at a negative transference, to say that “you seem dissatisfied without being able to say so, since you are missing sessions and come up with excuses”.

(T2)

In situations with experiences of negative transference, the therapists had experienced that TI could have the potential to repair ruptures in their therapeutic relationship. To refrain from TI in those situations was experienced as frustrating because of their fear of losing the alliance. Use of TI was regarded as more helpful in some therapies than in others: “It was obvious that it was more important to use it with some patients than with others. Sometimes, I remember feeling that the patient needed something that I could not give—a different type of contact” (T6). The therapist further described a subjective need in her countertransference when faced with these patients. This points to an aspect of therapy that was highlighted by some therapists; that is, the discrepancy between their subjective experience and the patient’s experience. Even though therapies without TI could be challenging for them as therapists, their experience was that the challenges were “mostly in their own heads,” and that the adolescents improved in both treatment groups. One comment was: “It is hard to say if it made a difference for the adolescents … The biggest difference was probably for me personally, but … I am still the same person (laughs)” (T5).

The therapists reflected on the implications that refraining from TI had for their therapies. One comment was: “I was continually interested in what it did to me as a therapist to not have the opportunity to work with transference. I think that was a great question, both personally and theoretically, about consequences this had for my practice” (T4). He shared a similar experience as the therapist above. Even though he felt more inhibited in therapies without TI, his feedback from the adolescents was that these therapies had been helpful as well.

For all the therapists, the concept of *transference* was an internalized theoretical framework in which they interpreted and made sense of the adolescents’ problems. This framework guided their therapeutic interventions even when there was no explicit transference intervention during therapy. One therapist made the following remark:

I have the theory with me no matter what. So, I did not find it that hard not to say much about our relationship even though I interpreted the transference inside of me. It tells me a lot about the patient, even if I don’t address it.

(T1)

The therapists described how they could not eliminate or suppress getting transference reactions or insights that they drew from their countertransference. As a different therapist said: “It is with me anyway, so it is just a question about how I use it” (T6).

*Subtheme (II): Relational interventions can be useful to analyze the patients’ lifeworld*

A theme that was commonly addressed concerned how the therapists could provide the adolescents with the same help in therapies without TI as they did in therapies with such interventions. A typical experience was that psychodynamic therapies without TI contained many of the same elements that can facilitate change. One therapist described it this way: “I still try to understand, I am curious and I am concerned with what they are dealing with [and] their experiences with other people” (T4). The therapists said their role and therapeutic attitude was constant in both therapy modes. Refraining from TI did not prevent them from being concerned with the adolescents’ experiences and trying to give them validation and affirmation. They could also address and analyze specific situations that the adolescents presented: “So, for example, if there was a conflict between two different desires that both were important, we could look at and analyze their inner conflicts and choices together” (T2).

Talking about specific situations in the adolescents’ everyday life was a common therapeutic strategy to help the young people gain insight and perspective. Most therapists described how therapies without TI revolved mostly around the adolescents’ daily lives: “… And then we analyzed that, what happened at school, what was going on at home, the crisis with the stepmother…” (T1).

Several therapists addressed issues related to providing insight into relational patterns in ways other than interpreting transference. A common intervention was to use relationships outside of therapy, when appropriate. Using RI was described as more useful in sessions in specific relationships that were well suited for giving the patient insight into a relational pattern that had been mentioned: “I used examples like ‘it might be like that with your friends too, or with your mother’ ... So, they brought something that was a problem out there into therapy and we talked about it” (T5).

Refraining from TI was described as more challenging in therapy sessions where the therapists experienced a transference phenomenon in the session. The comment below illustrates this:

I sort of had to think “what is it that the person struggles with that can be illustrated by something she has told me before, in relation to others.” It was kind of like “transference by proxy.” We’re really talking about us, but we pretend we are talking about someone else.”

(T2)

A common experience was that the adolescents were more concerned with their everyday lives than with the relationship between them and the therapist. To analyze other relationships was experienced as useful as they could talk about situations that the adolescents were right in the middle of. This could provide a chance to relate and react to these situations in a different way.

Some therapists felt the emotional temperature was lower in therapies without TI. One described the therapeutic relationship as more distant, and that it made it easier for her to distance herself from the patient. A different therapist described therapies without TI this way: “It does not get that intense, and that might be more comfortable for some” (T2). While some therapists described the emotional temperature as usually being lower in these therapies compared to therapies with TI, one therapist thought the therapeutic relationship usually became as close in both types of therapies:

I have thought a lot about that, whether using transference interventions affects how the therapeutic relationship develops. And I don’t think it does (laughs). My expectation was that it would, but as I practiced intervening without using transference, I experienced that something happened between us either way.

(T4)

Although, the therapist’s experience was that the therapeutic relationship could develop in a similar way in both therapy modes, that it took practice to adjust to intervening without the use of TI.

### 3.3. Key Theme 3: You Always Need to Adapt to the Particular Patient

The third key theme concerned when and with whom TI was suitable. Most therapists mentioned how therapeutic interventions always need to be adapted to the individual patient. Their reflections could be sorted into two different, but related themes: (1) the therapeutic interventions must be relevant to the therapeutic material, and (2) some patients might be better off without TI.

*Subtheme (I): The therapeutic interventions must be relevant to the therapeutic material*

Most therapists said there were therapies where TI was less suitable for helping adolescents with their issues. There were several situations where they experienced it was useful to refrain from using TI. One therapist noted that something must be activated in the transference for the interventions to be useful (T5). She said this was less frequent with patients with high relational functioning, and patients without personality problems. Another dimension that was addressed was the timing of therapeutic interventions. Some therapists said, when adolescents are in the midst of big emotional crises, it is best to refrain from TI to make room for the crisis the patient was going through. One therapist described a therapy with a girl that had depression that was related to the loss of one of her parents:

With her I thought it was more of a grief-trauma we had to work with, rather than to start talking about transference. I could always introduce like “how was it to tell this to me?” but in the situation she was in, with such a big loss, I am not sure it would be helpful.

(T1)

The therapist felt TI was less relevant to use in this particular therapy because of the timing and the situation her patient was in, i.e., in the midst of a big life crisis with high emotional pressure.

One therapist described some therapies where the adolescents mainly wanted to address specific solutions to life situations. In his experience, those patients could profit from therapies without TI: “I think that the patients … get a calmer therapy, but they might get more opportunity to think about their life out there with a so-called wise person” (T2). His experience was that these adolescents could obtain insight into their relational patterns by him (the therapist) pointing out patterns or tendencies that he saw that the patient had in other relations.

*Subtheme (II): Some patients might be better off without transference interventions*

Typical examples revolved around the experience that adolescents with some traits or issues were either better off without TI, or that therapists needed to be more careful with their use of TI in therapies with these patients. In particular, two types of issues were defined: (1) adolescents with mentalization difficulties and (2) adolescents they thought were especially vulnerable and/or had experienced relational trauma. Difficulties with mentalization, communicative skills, and reciprocity were mentioned as the main barriers to receiving, understanding, and profiting from TI:

One patient had a very reduced ability to mentalize, or to work that way. So, we ended up with more of a superficial chat, like how his week had been, how his days had been. It was a lot of repetition. Talking about emotions was difficult.

(T3)

One therapist emphasized that in her general experience, patients with reduced ability to mentalize were better off with therapies without TI. She described it as follows: “They can get quite scared by it. You have to adapt to the patient and what she needs, and you sense that quite quickly” (T6).

Some mentioned therapies where they experienced the patient as too vulnerable to take advantage of TI. One therapist experienced a patient drop out after a session with a particular transference intervention:

I was very reluctant about a transference intervention with her. Eventually, I did it and I think it became too tough for her, very tough and too difficult. I remember thinking “Oh no, now I got too close!” and after that she disappeared.

(T5)

When reflecting on that therapy in hindsight, the therapist described it as premature to comment and interpret the transference: “I am sitting there considering therapeutic interventions all the time, right. It was just as if I could feel it in the countertransference that it was not good for her that I addressed our relationship at that time.” While this therapist thought her patient could have profited from similar TI later in the therapeutic process, a different therapist had a patient that he thought was too vulnerable to profit from TI during the whole course of the therapy:

… She was really suffering and struggling with her past and was not ready to process her history in terms of abuse and such. Doing transference interventions with her was very difficult. She was depressed, but there was also a trauma aspect, of course. I felt I had to be very careful because she was so vulnerable.

(T3)

In both cases, the adolescents had experienced relational trauma. Both therapists addressed the need to adjust the therapy to the adolescents, whom they experienced as being more vulnerable because of their relational history and background.

## 4. Discussion

The present study aimed to explore how therapists experienced psychodynamic therapies with and without TI for adolescents with depression. The analysis resulted in three key themes. The therapists highlighted that TI are often useful, as they can strengthen the therapeutic relationship and contribute to increased insight and greater self-esteem. Therapies without use of TI could be experienced as limiting for the therapist; however, using RI was viewed as being helpful for analyzing the patients’ lifeworld. The therapists identified sub-groups of adolescent patients that they experienced were better off without TI and stressed the need to adapt the therapy to the particular patient.

### 4.1. How Do the Therapists’ Experiences Contribute to Existing Research on Transference Interventions?

Interpretation of the patient’s relationship to and experience of the therapist’s TI has been considered to be important because they are frequently used techniques in psychodynamic theory [60,73,74]. The therapists interviewed in this study highlighted how they needed to experience that something was activated in the transference for TI to be experienced as relevant and purposeful. The therapists experienced it as superfluous to use TI in therapy with adolescents that were relatively high-functioning or had no personality pathology. Higher relational functioning might suggest that less is activated in the transference [26,27,28,30]. Research on adults indicates that moderate levels of TI can be useful in therapy with patients who have personality problems, as well as those who have serious and chronic difficulty in establishing stable and meaningful relations. Moderate levels of TI also seem to have specific positive effects on the long-term functioning of patients with low relational functioning [28]. Similar analyses of adolescent patients are yet to be published, and different characteristics might be true for young people. The therapists in the present study highlighted several clinical issues and situations in therapy with adolescents that might suggest that the therapists need to be careful when they use TI or refrain from using TI. Adolescents who are in the midst of an emotional crisis or have experienced serious relational trauma, and adolescents who have a reduced capacity to mentalize were mentioned as patient groups that might benefit from therapies without TI. McCullough and colleagues [75] found that TI that were met with affective elaboration by the patient predicted positive outcomes in brief dynamic therapy. While the therapists generally perceived TI as useful to strengthen the therapeutic relationship and potentially prevent dropouts, one therapist experienced that a patient had dropped out after the use of a transference intervention. This suggests that it is ultimately the quality of TI that is most likely to predict their effectiveness [74].

How to best tailor interventions in STPP has been debated [76]. Christogiorgos et al. suggested that TI in STPP should focus on positive transference and on work in the “here-and-now” [77]. Others believe that exploration of the transference to the external environment (e.g., school or mental health service) should be the main focus in STPP, because this would help adolescents reflect on the ways they relate to the external world [78]. The therapists in the present study experienced a range of TI as being useful in STPP. Their general experience was that use of TI could strengthen the therapeutic relationship. TI was also perceived as potentially making the therapeutic relationship more vulnerable. This was suggested to have in mind, especially in short-term therapy and when it comes to the issue of ending therapy. A process study found that TI around endings may trigger different types of reactions, which might reflect ways that adolescents use to deal with the anxiety around ending and separation from the therapists [76]. The authors conclude that the focus on transference should be paced and adapted in a flexible way, based on the particular patient and his/her responses when therapy is coming to an end [76]. Many clinicians work in public healthcare and are guided by mental health policies and conditions that limit the amount of time available for treatment [79]. This suggests that the available timeframe is an institutional dimension of therapy that therapists need to be mindful of when considering how to tailor their interventions in therapy with adolescents.

### 4.2. Relational Interventions Can Be Useful When Working with Adolescents Because of Their Developmental Stage

Therapists in our study considered RI focused on interpersonal relationships as useful for addressing and analyzing adolescents’ lived experiences. The young people they had in therapy were most concerned about the events and situations that were going on in their everyday lives. Adolescents need to undertake several developmental tasks to make a successful transition into adulthood, including sexual maturity, emotional development, thinking capacity, forming mutually close and supportive friendships, and renegotiating relationships with adults in parenting roles [80,81]. The therapists experienced that it was important for the adolescents to have a therapeutic space to address life situations, and that RI could be useful to look at and simultaneously analyze the patient’s conflicts between different desires. This can be particularly relevant with adolescents when considering that negative changes in interpersonal adjustment (e.g., deterioration in family relationships or withdrawal from peer relationships) are among the most common clinical features of adolescent depression [1,82]. This withdrawal might prevent a young person from working on important developmental tasks in close relationships with family and peers [80].

A qualitative study with adolescents from the FEST-IT found that therapists’ questions about what adolescents felt in different situations were considered especially helpful in the process of opening up and making them feel acknowledged by the therapist [45]. The adolescents experienced improvement by talking about emotions and thoughts, and by doing so, they got to know themselves better. Alternative ways to handle their problems also became clearer by exploring their own thoughts and actions [45]. All the adolescents said it was helpful when therapy became practical and focused on specific challenges in their everyday life. Some even described this as the most important element in therapy, and that getting explanations for their situations and answers to what was happening to them was crucial for their improvement [45]. This is in line with another qualitative study with adolescent patients, in which one of the most important dimensions for them was that “the therapy helped them make their experiences understandable and meaningful” [83]. The adolescents in the latter study described that an important aspect in the process of building trust and safety in the therapy situation was the experience that the therapist could support their search for meaning in their experiences [83]. These descriptions from therapy suggest that use of relational interventions can facilitate a helpful conversation about experiences and situations that adolescent patients are in the middle of. This conversation has the potential to not only make them more aware of themselves, but promote specific improvements in their everyday life as well [45]. Patients and therapists represent complementary positions in the therapy room, and hence, they emphasize different aspects in their descriptions of therapy. The adolescents were more practically oriented, whereas the therapists drew on psychodynamic theories to make sense of experiences in therapy. Both accounts indicate that interventions that focus on the patients’ interpersonal relationships can contribute to insight into adolescents’ experiences; thus, they provide an opportunity to relate and react to situations in new ways, with peers, relatives, teachers, and other important relations in the young person’s life.

Little empirical research has been done on the actual techniques that are used in psychodynamic therapy with adolescents. Process analyses of the psychodynamic treatment arm in the IMPACT study have revealed that STPP therapists focused less on certain classical psychoanalytic features, such as exploration of the relationship between the therapist and patient, exploration of dreams and fantasies, and past and repetitive patterns in relationships. Interventions, such as working with the adolescent’s current preoccupations and interpersonal relations, and helping them to express and understand their feelings and experiences were featured more in the therapies [58,84]. This corresponds with therapist and patient accounts from the FEST-IT and adds to our knowledge about how adolescent STPP is conducted.

### 4.3. Therapists’ Perspectives Are Essential in Clinical Research

An important finding of this study is that the therapists, to a greater or lesser extent, experienced it as challenging to refrain from TI, especially in sessions where they experienced a transference phenomenon. This experience of being constrained or limited is not necessarily related to negative patient outcomes. The concept of professional self-doubt and humility has begun to receive attention lately [85,86]. A high level of professional self-doubt, in combination with a high degree of self-affiliation as a person, has been found to be particularly fruitful, while the combination of little professional self-doubt and high positive self-affiliation is not [86]. These self-concept states are assumed to be communicated through the therapists’ in-session behavior [86]. Some therapists in the present study experienced a discrepancy between their own experience of the therapy and the adolescent’s change during therapy. That there can be a discrepancy between how therapists and patients experience therapy is supported by research showing low correlations between patients’ and therapists’ evaluations of the dimensions of therapy, e.g., evaluations of therapeutic alliance [87,88]. However, the therapists’ experience of being constrained is important to take notice of, as it can affect their ability for ongoing moment-to-moment responsiveness in their communication with patients [89].

Therapists’ perspectives and experiences are essential for identifying barriers to implementing new knowledge in clinical practice. The therapists’ accounts in our study showed that they found it challenging to refrain from using familiar techniques, such as TI in psychodynamic therapies. This can affect their confidence in the treatment method, also known as therapy allegiance [90]. Our findings indicate that training and supervision in psychodynamic therapy with RI (instead of TI) can be useful to enhance the therapists’ confidence and belief in the therapy format. This can enhance the experience of being able to help, which in turn is associated with the experience of being more free and creative and less inhibited [91]. The role of theory in therapy has been the subject of attention in several studies, see, for example, References [49,92,93]. The therapists’ reflections about TI in this study suggest that the concept of transference is a theoretical framework that they can use to understand and make sense of their adolescent patients, even in therapies that do not use TI. The tension between acknowledging the important role of theory while, at the same time, wishing to remain close to the experience of the patient rather than interpreting the experience through a fixed theoretical lens was documented by a study with senior therapists [49]. Adolescent patients emphasize the importance of the therapist appearing to be comfortable with being a therapist—experienced and competent and talking with confidence—and providing guidance and support [45,83]. The therapist effect is recognized as a durable and robust phenomenon that explains approximately 5% of the variance in outcomes, on average [94]. One hypothesis is that the differential effectiveness among therapists is related to the way individual therapists carry out selected elements of therapy and that these elements differ or overlap among therapists [94]. The techniques, and how they are performed in therapy, are not insignificant [91]. It is through specific techniques that the therapist, together with the patient, co-creates a healing relationship that can produce therapeutic change [95]. For this very reason, knowledge about how therapists experience different treatment modalities and use different interventions in therapy is needed.

There are several limitations related to the exploratory intent and contextualized nature of the present study. The sample in this study was limited to six participants. Out of eight therapists who were contacted, two declined to participate. According to the ethical protocol, the invited therapists were free to refuse to take part without stating any reason. A consequence of this practice was that we have no information regarding why some of the therapists refused to participate in the study, and if they differed from those who volunteered to be interviewed in a way that would have had a significant effect on the final results. Furthermore, some therapy processes were conducted several years back in time, which might have affected the therapists’ memory and perception of the therapies. The interpretation of the data must take into consideration that the experiences did not necessarily reflect what actually happened in therapy [65], but how the therapists understood and made sense of their experiences. That the participants tried to make sense of processes that included a third party (the adolescents) adds a layer to the interpretative process. Finally, the therapists were interviewed based on their experiences participating in an RCT where they had to either use TI, or refrain from using TI [59]. This restriction was due to the experimental design, and it may not be directly transferable to a clinical setting.

## 5. Conclusions

The present study described the clinical experiences of therapists regarding two different psychodynamic treatment modes for adolescents with depression: therapy with and without TI. The results enhance our knowledge of the significance of the therapist’s actions in adolescent therapy. Furthermore, the therapists highlighted issues that are important for identifying barriers to incorporating new knowledge into clinical practice. This implies that in order to implement the research results from the RCT into clinical practice, training and supervision, such as peer-supervision in groups, can be useful for therapists to get enhanced confidence in working differently. Further research is needed to enhance our knowledge of how interventions and interaction structures play out in adolescent therapy. Triangulation of data [39], such as also exploring the content of TI and RI used in successful therapies, can provide useful information regarding “how to practice” in therapies with adolescents with depression.

## Figures and Tables

**Table 1:** How do therapists experience psychodynamic therapies, with and without transference interventions, for adolescents with depression?

**Key theme 1: Transference interventions are often useful**
Subtheme (I): Working with transference strengthens the therapeutic relationship. Subtheme (II): Transference interventions can contribute to increased insight and greater self-esteem.
**Key theme 2: Therapies without transference interventions can be challenging for the therapist, but still helpful for the patient**
Subtheme (I): Refraining from transference interventions can be experienced as a limitation for the therapist. Subtheme (II): Relational interventions can be useful to analyze the patients’ lifeworld.
**Key theme 3: You always need to adapt to the particular patient**
Subtheme (I): The therapeutic interventions must be relevant to the therapeutic material. Subtheme (II): Some patients might be better off without transference interventions.
